# B-mode ultrasound for the assessment of hepatic fibrosis: a quantitative multiparametric analysis for a radiomics approach

**DOI:** 10.1038/s41598-019-45043-z

**Published:** 2019-06-18

**Authors:** Julia C. D’Souza, Laith R. Sultan, Stephen J. Hunt, Susan M. Schultz, Angela K. Brice, Andrew K. W. Wood, Chandra M. Sehgal

**Affiliations:** 1 Ultrasound Research Lab, Department of Radiology, University of Pennsylvania, Philadelphia, PA USA; 2 Penn Image-Guided Interventions Lab, University of Pennsylvania, Philadelphia, PA USA; 3 University Laboratory Animal Resources, University of Pennsylvania, Philadelphia, PA USA; 4 Department of Pathobiology, School of Veterinary Medicine, University of Pennsylvania, Philadelphia, PA USA

**Keywords:** Liver cirrhosis, Preclinical research

## Abstract

Hepatic fibrosis and cirrhosis are a growing global health problem with increasing mortality rates. Early diagnosis and staging of hepatic fibrosis represent a major challenge. Currently liver biopsy is the gold standard for fibrosis assessment; however, biopsy requires an invasive procedure and is prone to sampling error and reader variability. In the current study we investigate using quantitative analysis of computer-extracted features of B-mode ultrasound as a non-invasive tool to characterize hepatic fibrosis. Twenty-two rats were administered diethylnitrosamine (DEN) orally for 12 weeks to induce hepatic fibrosis. Four control rats did not receive DEN. B-mode ultrasound scans sampling throughout the liver were acquired at baseline, 10, and 13 weeks. Computer extracted quantitative parameters representing brightness (echointensity, hepatorenal index) and variance (heterogeneity, anisotropy) of the liver were studied. DEN rats showed an increase in echointensity from 37.1 ± SD 7.8 to 53.5 ± 5.7 (10 w) to 57.5 ± 6.1 (13 w), while the control group remained unchanged at an average of 34.5 ± 4.5. The three other features studied increased similarly over time in the DEN group. Histologic analysis showed METAVIR fibrosis grades of F2-F4 in DEN rats and F0-F1 in controls. Increasing imaging parameters correlated with increasing METAVIR grades, and anisotropy showed the strongest correlation (ρ = 0.58). Sonographic parameters combined using multiparametric logistic regression were able to differentiate between clinically significant and insignificant fibrosis. Quantitative B-mode ultrasound imaging can be implemented in clinical settings as an accurate non-invasive tool for fibrosis assessment.

## Introduction

In the United States, chronic liver disease and its end state of cirrhosis represent the fifth leading cause of death for people aged 45 to 54 years and the 12th leading cause of death overall, independent of associated deaths from liver cancer and complications of liver disease^1^. In the last two decades, mortality in the United States due to cirrhosis increased by 65%, with a specific increase in deaths of younger people ages 25–34^2^. The most common causes of cirrhosis are alcoholic liver disease, hepatitis C, and hepatitis B. Additionally, cirrhosis due to metabolic syndrome-induced non-alcoholic fatty liver disease is increasing. In all of these conditions, chronic inflammation of the liver leads to hepatic fibrosis and scarring, which progresses to cirrhosis. Given cirrhosis’ growing burden of disease, and its preventability through treatment and reversal of early stages of fibrosis, an easily-accessible assessment of fibrosis is imperative^3–5^.

The gold standard for assessing fibrosis is hepatic biopsy with subsequent analysis by pathologists. Biopsy is performed percutaneously or transjugularly, and the level of fibrosis is graded by pathologists using a semi-quantitative system such as the METAVIR system^6^. However, there are limitations to hepatic biopsy, including complications of the invasive procedure, sampling error, and intra- and interobserver variability^6,7^. These limitations highlight the need for alternative methods of assessing fibrosis that are noninvasive, minimize sampling error, and are objective. Proposed alternatives include radiologic tests and laboratory tests for hematological, biochemical, and direct markers of fibrosis^8,9^. Transient elastography, or FibroScan, has emerged as a promising sonographic technique to measure the elasticity of the liver, which becomes less elastic as fibrosis progresses^8,9^. While a meta-analysis found a mean area under the receiver operating characteristic curve (AUROC) of 0.94 for cirrhosis, the accuracy of differentiating clinically significant fibrosis at lower levels (F0/1 vs. F2/3/4) was weaker (AUROC = 0.84)^10^. Similarly, accuracy of ARFI in diagnosing fibrosis drops from AUC of 0.92 for cirrhosis to 0.87 for clinically significant fibrosis of F2 or greater^11^. Thus, improvement of tools assessing hepatic fibrosis can be especially helpful to monitor fibrosis early its course.

Toward this goal, the computerized analysis of liver features on B-mode ultrasound (US), which is widely available and used commonly in the clinic, could help to noninvasively characterize the progression of fibrosis. While US examinations have been used to detect fatty liver disease in humans^12–17^, less attention has been given to its potential for monitoring hepatic fibrosis. Studies focusing on hepatic fibrosis have largely assessed qualitative structural changes on ultrasound that are visible by eye^18^. However, computerized analysis of ultrasound images may provide measurements of liver fibrosis on a more continuous scale using unbiased quantitative imaging biomarkers. This correlation of quantitative radiographic measures with clinical states and outcomes is utilized in radiomics. Thus far, the use of radiomics in US has likely been limited by the variability of US acquisition and data extraction^19^. However, the advancement of standardization techniques such as the hepatorenal index to normalize tissue echointensity within a subject^20,13^ and the development of automated parameter maps to standardize analytical outputs support the utility of US radiomics^19^. Applying computerized analysis to the liver may allow for measurement of fibrosis from common, standard B-mode abdominal examinations using quantitative continuous measures.

In this study we image fibrosis as it develops over time, using a diethylnitrosamine (DEN)-induced rat model of liver injury that closely mimics human inflammatory liver disease. DEN produces primary metabolic activation and DNA alkylation, leading to hepatic necrosis, inflammation, fibrosis, and eventual carcinogenesis in a controlled setting not possible in human subjects^21–23^. We utilize quantitative analysis of computer extracted parameters from B-mode US images to widely sample and noninvasively characterize the progression of hepatic fibrosis.

## Results

### The effect of diethylnitrosamine is demonstrated by changes in weight over time and confirmed by histopathologic analysis

The initial weight of all animals was between 350–400 g. The weights of the 22 rats in the DEN cohort (396 g ± SD 52 g) lagged below control animals’ weights (445 g ± 32) by the second week of the diet (*p* = 0.003 at day 17 of diet) and remained lower for the remainder of the experiment. The final weight of DEN-exposed group was 447 g  ± 80; the final weight of controls was 633 g  ± 43 (*p* = 0.0005). The DEN-exposed animals developed liver disease and multiple foci of hepatocellular carcinoma (HCC), seen on gross pathology and histologic analysis. These changes were not seen in the control cohort.

### Ultrasound images of DEN-exposed livers reveal changes in quantitative measures of brightness and variance over time

**Figure 1:**
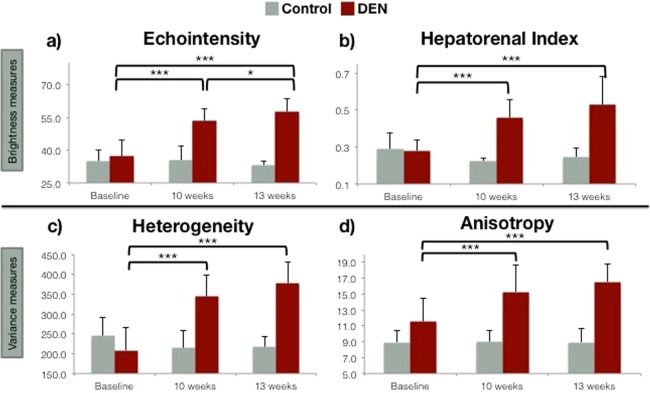
Ultrasound measures of texture increase in DEN cohort compared to healthy liver over time. A common trend emerged in the observations of each of the four sonographic features ((**a**) echointensity, (**b**) hepatorenal index, (**c**) heterogeneity and (**d**) anisotropy) In the control and treated groups, there were no statistically significant differences in the observations made at baseline (time = 0 weeks) and, further, the values for the control group did not change over time. There were, however, significant increases in the values of each the sonographic parameters at 10 and 13 weeks in the DEN treated rats.

When scanned at baseline before exposure to DEN, rats in the DEN cohort showed statistically equal values to the control cohort for all US parameters: echointensity, hepatorenal index (HRI), heterogeneity, and anisotropy (t-tests *p* = 0.62, 0.81, 0.24. 0.10, respectively; Fig. 1). Echointensity and hepatorenal index represent the echogenicity or brightness of the ultrasound images, with greater scattering and reflection of the ultrasound waves by the tissue leading to increased brightness. Echointensity is the mean brightness of the parenchyma analyzed in regions of interest (ROIs), and HRI normalizes that brightness to the right renal cortex intensity. As the DEN rats continued their diet, the liver echointensity increased (repeated measures ANOVA *F*_*2,39*_ = 59.6, *p* < 0.0005 for time points of 0, 10 and 13 weeks). Beginning at a baseline value of 37.1 ± SD 7.8, echointensity increased to 53.5 ± 5.7 at 10 weeks (*p* < 0.0005) and continued to increase to 57.7 ± 6.1 at 13 weeks (*p* < 0.0005, 0 to 13 weeks; *p* = 0.046, 10 to 13 weeks). In contrast, the control group showed no significant change in echointensity over time and showed a mean value of 34.6 ± 4.5 (*F*_*2,6*_ = 0.27, *p* = 0.77). Hepatorenal index rose from 0.28 ± 0.06 to 0.46 ± 0.10 at 10 weeks (*p* < 0.0005) and 0.53 ± 0.15 at 13 weeks in DEN rats (*p* < 0.0005, 0 to 13 weeks; *p* = 0.052 from 10 to 13 weeks; ANOVA *F*_*2,39*_ = 29.18, *p* < 0.0005). In the control rats, HRI remained an average of 0.25 ± 0.05 (*F*_*2,6*_ = 1.44, *p* = 0.31).

Similarly, in the DEN cohort the US measures of tissue variance increased over time (Fig. 1c,d). Heterogeneity is the standard deviation of brightness within ROIs and represents the local variance or speckle of the tissue. Anisotropy, used here to describe the variance in echogenicity between different spatial regions of the liver, is calculated as the standard deviation of brightness among ROIs in all planes imaged. Heterogeneity increased over time in the DEN-exposed rats but stayed constant at 225.7 ± 37.6 in control rats (*F*_*2,39*_ = 64.30, *p* < 0.0005 for DEN cohort; *F*_*2,6*_ = 0.57, *p* = 0.58 for controls). In the DEN cohort, heterogeneity rose from a baseline of 208.7 ± 58.3 to 344.6 ± 52.9 at 10 weeks and 376.8 ± 54.9 at 13 weeks (*p* < 0.0005, 0 to 10 weeks and 0 to 13 weeks; *p* = 0.06, 10 to 13 weeks). Anisotropy increased in a pattern similar to local heterogeneity. In control rats, anisotropy remained low over time at an average 8.96 ± 1.55 (*F*_*2,6*_ = 0.00, *p* = 0.9889). DEN-exposed rats’ anisotropy increased: baseline was 11.6 ± 3.0, statistically equal to the controls’ baseline (*p* = 0.10). Anisotropy in the DEN cohort then rose to 15.3 ± 3.4 at 10 weeks and 16.6 ± 2.2 at 13 weeks (ANOVA *F*_*2,39*_ = 29.18, *p* < 0.0005; 0 to 10 weeks, *p* < 0.0005 and 0 and 13 weeks, *p* = 0.18, 10 and 13 weeks).

**Figure 2:**
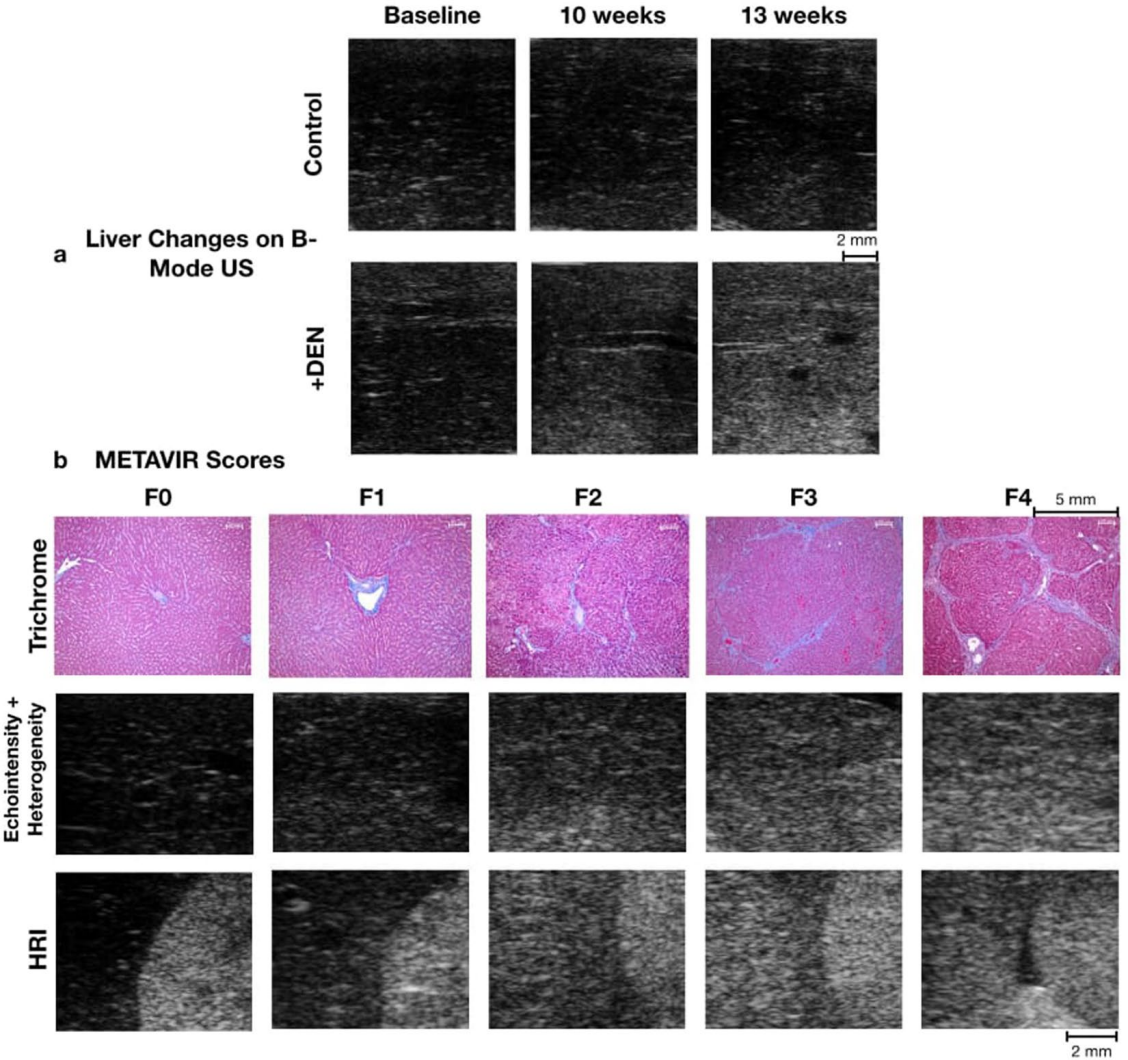
Sonographic and histologic findings. (**a**) Imaging changes over time: In B-mode ultrasound images of the control group, the liver was uniformly and consistently hypoechoic for the duration of the experiment, whereas in the DEN treated rats the liver over time became progressively more hyperechoic and its echogenic texture became grainier. (**b**) Tissue and imaging at varying severities of fibrosis: Trichrome-stained histologic images of the liver of the control and treated groups after sacrifice showed a range of pathologic change. Using the METAVIR grading system, collagen, staining blue, is only present in minimal amounts in the normal liver (F0, F1); as hepatic fibrosis increases in the DEN-treated rats, the amount of collagen increases outwardly from the porta and forming septae (F2, F3); eventually bridging fibrosis and cirrhosis occur (F4). US images of liver parenchyma from 13-week imaging: The greater values of echointensity and heterogeneity can be seen by brighter and more speckled parenchyma in more severe fibrosis. For HRI, the brightness of the right renal cortex and liver parenchyma become more similar as fibrosis progresses and are nearly equal in F4.

Changes in brightness and variance of the liver parenchyma are shown in Fig. 2. The control rats remain dark with low speckle. The DEN cohort becomes brighter by the 10- and 13-week time points, demonstrating the increase in echointensity. The heterogeneity, which may be noted as speckle, is seen as grainier texture in the DEN cohort. For HRI, the brightness of the right renal cortex and liver parenchyma become more similar as fibrosis progresses and are nearly equal in F4. While visual effects are seen with echointensity, heterogeneity, and HRI, the anisotropy is difficult to appreciate visually because it measures fluctuation in the gray levels from multiple regions and images throughout the liver.

The intra-observer analysis showed no significant difference in echointensity, heterogeneity, or HRI at 0, 10, or 13 weeks (p > 0.05 for each time point for each measure, paired Student’s t-test). Though there were some statistical differences in anisotropy between analyses, the same pattern of increase was present on both analyses. The values from both analyses and the p-values for the intraobserver analysis are shown as Supplemental Information (SI Table 1).

### Portal vein and inferior vena cava diameters remained constant over time, while the right hepatic vein diameter increased

The portal vein diameter was 1.71 mm ± 0.26 in controls and 1.70 mm ± 0.28 mm in the DEN cohort (*p* = 0.68) and did not change over time in control or DEN cohorts (*F*_*2,6*_ = 2.79, *p* = 0.16; *F*_*2,39*_ = 1.87, *p* = 0.18, respectively). Similarly, the IVC diameter stayed constant over time at an average of 4.07 mm ± 1.44. However, while the right hepatic vein stayed constant at 1.17 mm ± 0.37 in the control cohort (*F*_*2,6*_ = 0.33, *p* = 0.70), it increased over time in the DEN cohort (*F*_*2,24*_ = 6.17, *p* = 0.008). Right hepatic vein diameter rose from a baseline of 1.16 mm ± 0.33 to 1.30 mm ± 0.28 at 10 weeks and 1.62 ± 0.44 mm at 13 weeks (*p* = 0.002, 0 to 13 weeks; *p* = 0.02, 10 to 13 weeks). There was a trend toward significant difference between the DEN and control right hepatic veins at 13 weeks (*p* = 0.066).

### Histopathologic analysis showed a spectrum of METAVIR grades of fibrosis between DEN and control cohorts

Scoring of multiple samples of each rat’s liver by a veterinary pathologist showed two control rats with a highest grade of F0 in any section. The other two control rats showed F1 as the highest grade present. Of note, control sections with F1 as opposed to F0 were accompanied by an acute inflammation and multifocal necrotizing hepatitis more consistent with an infection than the chronic inflammation, fibrosis, and nodular regeneration seen in DEN-exposed rats. The DEN rats all showed fibrosis with a METAVIR grade of F2 to F4. This spectrum of fibrotic changes is demonstrated in Fig. 2. The width of fiber bundles measured on trichrome-stained images of F2-F4 rats ranged from 7.60–80.21 micrometers, with a median of 18.83 micrometers.

Gross pathology of DEN-exposed animals showed varying nodularity and counts of lesions typically appearing white and solid with variable levels of hemorrhage and liquefaction. Cellular and architectural changes included multifocal to extensive hepatocellular hemorrhagic necrosis, lymphoplasmacytic infiltrate with few neutrophils, and portal and septal fibrosis with and without formation of nodules. Additionally, the livers showed multiple foci of karyomegaly, vacuolated cytoplasm, and lesions of hepatocellular carcinoma with foci of necrosis and hemorrhage. Neither DEN-exposed nor control rats showed notable steatosis.

### Scoring of fibrosis using the METAVIR scale correlates with numeric values of US analysis and separates rats into distinct populations

**Figure 3:**
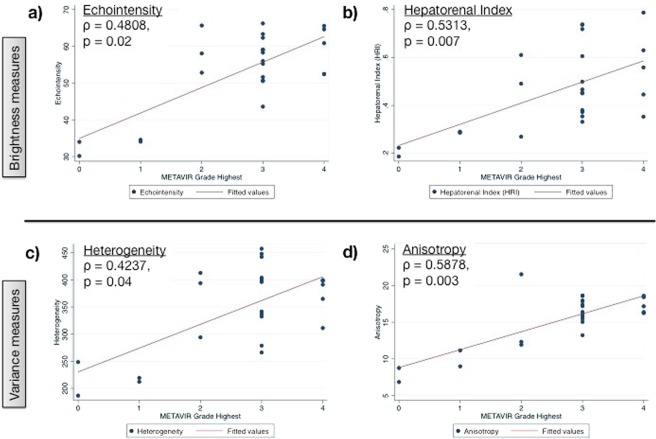
Spearman correlations between histologic METAVIR grade and multiparametric sonographic features. The level of fibrosis (METAVIR) was correlated with the numeric values of sonographic textural features (time = 13 weeks; (**a**) echointensity, (**b**) hepatorenal index, (**c**) heterogeneity and (**d**) anisotropy). Samples with insignificant fibrosis (F0, F1 from the control group) showed lower quantitative measures on ultrasound. There were positive correlation coefficients between the increasing level of fibrosis (F2 - F4 from the DEN group) and increasing ultrasound measures. Note the strongest correlation between anisotropy (variance between regions) and the progression of fibrosis, indicating an uneven pattern of development.

Severe grades of fibrosis by METAVIR criteria corresponded with high sonographic measures of fibrosis. This relationship is demonstrated by nonparametric Spearman regressions showing increasing ultrasound values with increasing METAVIR grade (Fig. 3). The highest echointensity level observed (67.1) corresponded to a METAVIR grade of F4 (cirrhosis) while the lowest echointensity level (54.3) in the DEN cohort showed less fibrotic change and corresponded to a METAVIR grade of F2. The Spearman rho correlation coefficients for echointensity, HRI, heterogeneity, and anisotropy were 0.48, 0.53, 0.42, and 0.58, respectively. All correlations were significant (p < 0.04).

**Figure 4:**
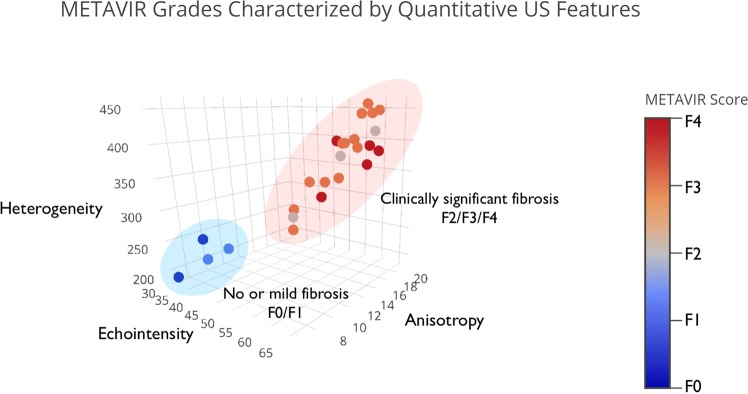
Multiparametric ultrasound analyses separate METAVIR scores into two populations. In this three-dimensional plot, the mean values of echointensity, heterogeneity and anisotropy for each rat in the treated and control groups are displayed, with METAVIR score indicated by marker color and color bar. Note the clear separation of the METAVIR populations into two groups: no or mild fibrosis (F0, F1) and clinically significant fibrosis (≥F2).

Additionally, the four features were used in combination to characterize the varying severities of fibrosis. An ordinal multiparametric logistic regression using all parameters was conducted, and stepwise backward selection included echointensity, heterogeneity, and anisotropy. HRI was excluded by this selection, most likely due to redundancy stemming from its relation to echointensity. The model was significant with a chi-squared = 23.75 significant at p < 0.00005. The model found a cut-point for prediction between F1 and F2 to have an odds ratio of 11.52 (standard error 3.79). When echointensity, heterogeneity, and anisotropy were plotted for each rat, the subjects separated into two distinct populations corresponding to the cutpoint between F1 and F2 (Fig. 4). The first population included rats with no or mild fibrosis (F0, F1), and the second population included rats with clinically significant fibrosis (≥F2).

The F0/F1 rats’ sonographic parameters at 13 weeks were found to agree with the DEN cohort’s baseline healthy tissue (p > 0.05, Supplemental Information Table 2), thereby suggesting that the control group is representative of clinically insignificant fibrosis demonstrated by a larger sample of healthy tissue. Additionally, the mean values and standard deviations of each feature for the DEN and Control groups were used to verify the sample size for the F0-F1 and F2-F4 groups (alpha = 0.05, power = 0.80). Anisotropy required the largest sample size (14 DEN rats +3 control rats), with other features requiring smaller sample sizes. Thus, the sample size selection of 22 DEN rats +4 control rats is appropriately powered to distinguish the clinically insignificant fibrosis and clinically significant fibrosis groups.

## Discussion

Cirrhosis is an increasing cause of death worldwide and in the United States. In addition to the traditional demographic of 45–55-year-olds, recent analyses show a 10.5% annual increase in mortality in 25–34-year-olds in the US^2^. This demonstrates the growing impact of cirrhosis on many populations with notable implications for public health. Preventing the development of cirrhosis and its complications, such as peritonitis, sepsis, variceal bleeding, ascites, and hepatic encephalopathy, will improve the outcomes of patients with chronic liver disease. Advancement in current methods of assessing and monitoring changes hepatic fibrosis will be important for patient management as well as for studies developing antifibrotic drugs^24^.

A noninvasive, broadly sampling, and quantitative assessment of hepatic fibrosis would reduce the need for hepatic biopsy with the attendant problems of procedural complications, sampling error, and subjectivity of a qualitative assessment of pathology. Previous reports of using ultrasound to detect cirrhosis showed inadequacy as detection relied on morphologic features accompanying end-stage cirrhosis, such as liver and spleen length, liver surface nodularity, hepatic vein straightness, and hepatic vein wall echogenicity^12,15^. Additionally, qualitative assessments of hepatic fibrosis have been used to characterize the echotexture as being fine, mildly coarse, coarse, and highly coarse^17^. In previous qualitative assessments of hepatic steatosis, operator dependency and subjectivity led to high inter- and intra-observer variability^25^. The accuracy of such evaluations may be improved through quantitative assessments of the images.

In this study, quantitative ultrasound features changed with verifiable progression of rats’ chronic liver disease and hepatic fibrogenesis. Specifically, DEN uniformly led to reduction in the weight gain of DEN-exposed rats, consistent with previous observations of this model^26^ and created progressive hepatic fibrosis verified by histologic evaluation. The four computer-extracted features studied in the B-mode US images (echointensity, HRI, heterogeneity, and anisotropy) consistently increased over time as hepatic disease progressed. Echointensity, or mean brightness of the liver parenchyma, increased significantly by the 10-week timepoint. Initial low levels of echointensity and speckle noise in the control and baseline scans are likely due to the homogenous nature of the tissue which lacks significant collagen and strong ultrasound scatterers. As fibrosis develops, collagen and an inflammatory cell infiltrate appear and could both act as acoustic scatterers to cause ultrasound wave backscatter and increase speckle brightness^17^. While brightness has been studied as a feature of steatosis, histologic assessment validated that the changes in US echointensity seen here occurred in a steatosis-free progression of fibrosis. The increase in mean brightness was also detected when normalizing the hepatic echointensity to that of the right renal cortex, or the hepatorenal index (HRI)^13^. HRI improved radiologic-pathologic correlation with the METAVIR grade of fibrosis (ρ = 0.48 in echointensity to 0.53 in HRI). This normalization technique may be used to minimize variation in instrumentation settings between a subject’s examinations and give the technique greater utility in retrospective clinical studies and in clinical practice^20^.

The two measures of variance studied, heterogeneity (local variations in echogenicity found within ROIs) and anisotropy (regional variations in echogenicity found between ROIs), also increased over time. These increases likely correspond to generalized formation of collagen septae and align with previous qualitative descriptions of an increased coarse texture in fibrotic livers^27^. In the fibrotic liver, the acoustic interfaces increase and the acoustic impedance difference between fibrotic tissue and hepatocytes becomes larger^28^. This leads to the observed coarse, echogenic pattern, with variation between areas with frequent collagen septae that scatter US and areas lacking septae. Regional differences demonstrated by increasing anisotropy may indicate variable severity of disease throughout the liver lobes. This is possibly related to variable distribution of portal blood flow and variable toxin distribution and metabolism. Furthermore, this calls attention to the fact that mixed levels of disease coexist. Understanding the most severe level of a patient’s disease as well as the amount of healthy liver reserve based on imaging could be helpful clinically. Aside from parenchymal markers, the increasing right hepatic vein diameter may be due to liver tissue contraction causing venous expansion rather than elevated luminal hepatic venous pressure, and serve as an easily quantifiable marker of progressing chronic liver disease. These imaging biomarkers may serve as an early exploration into the use of quantitative ultrasound biomarkers and radiomics in ultrasound.

This set of imaging biomarkers may also provide a platform for the development of artificial intelligence and radiomics in clinical ultrasound, by relating biomarkers from imaging datasets with pathologic METAVIR staging. Beyond looking at each sonographic feature on its own, we grouped measures of brightness and variation together to characterize the stages of fibrosis. When analyzing echointensity, heterogeneity, and anisotropy simultaneously, two distinct populations of METAVIR grades were distinguished. The first included F0-F1 (no fibrosis to portal fibrosis), which is considered clinically insignificant and does not warrant treatment in the case of viral disease. The second population represents “clinically significant fibrosis” comprising F2-F4: minimal septae, numerous septae, and bridging fibrosis (cirrhosis). Clinically, these patients require more active management. Within these groups there is some overlap between METAVIR categories, which may reflect the temporal and spatial heterogeneity of fibrosis between lobes^29,30^. However, the distinction between clinically significant and clinically insignificant disease using these three imaging biomarkers indicates that both brightness and variance measures are useful in characterizing the development of fibrosis using ultrasound. The implementation of a multiple-biomarker approach using these quantitative features in isolation or in combination with additional imaging features, serum biomarkers, and clinical information may ultimately provide the optimal predictor of clinical outcome.

In this study, imaging was performed at frequencies greater than those used for clinical scanning (21 MHz versus 3–7 MHz). Details of the tissue microstructure in the echosignal depend on the ultrasound frequency of the imaging pulse^31–34^. Higher frequency provides higher spatial resolution^35^ and greater backscatter^36–38^. The use of a higher frequency potentially provides more detailed information on tissue microstructure than is possible with current clinical imaging systems. This benefit, however, comes at the expense of shallower penetration due to greater attenuation of ultrasound.

In addition to frequency, the tissue microstructure, including the tissue composition, fiber size, cell density, and cell size, also affect the ultrasound backscatter^34^. The architecture of the liver is complex and consists of hepatocytes organized into larger units called acini, which develop a significant component of collagen fibers during fibrosis^39^. In humans compared to rats, hepatocytes are roughly equivalent in size^40–42^, acini are twice as large^39,43^, and the maximum fiber thicknesses^44^ are twice of those reported in this study (Table 2 Supplemental Information). These differences in microstructure between the two species could influence the echogenicity. On one hand, the lower frequency used in clinical scanning would result in lower scatter, but on the other hand, the larger scale of the tissue microstructure in humans would create greater scatter. These opposing effects likely have a complex interplay on the net echogenicity, which is not well understood for complex media such as the liver microstructure’s multiple scales of acoustic scatterers (cells, fibers, fiber-encircled acini and nodules). The larger scale of human tissue components may explain the ability of clinical gray scale ultrasound to observe textural and echogenic changes previously^12,16,27,28^. Further validation by side-by-side comparison of high and low frequency ultrasound in human subjects would provide better insight into whether the studied tissue changes are apparent using clinical scanners.

The benefits of B-mode US include the widespread availability of imaging systems, its capability to assess hepatic parenchyma concurrently with ascites, hepatic masses, or other abdominal indications, and its ability to image a large liver volume to reduce sampling error. By further standardizing and automating ROI selection, measurement could occur automatically in clinical abdominal ultrasound examinations to assess liver health. This easily accessible, noninvasive tool could improve longitudinal monitoring of disease progression or response to therapy while avoiding the need for multiple biopsies.

Limitations of our study include the possible changes in fibrosis between 13-week imaging and sacrifice and the use of a controlled model of fibrosis, which may not account for concurrent liver pathology from varying etiologies. Previous studies on nitrosamine-induced fibrosis show mix of fibrosis progression, regression, and stability after stoppage of toxin administration. While some evidence shows a reduction in the collagen after stopping the toxin^45^, other studies showed progression in fibrosis between 2–24 weeks after stoppage^46^. This progression was hypothesized to be due to genotoxic or metabolic insults to hepatocytes during toxin administration. Variation due to either of these processes could affect the correlation between US imaging and METAVIR staging in this study, potentially causing some of the variability observed. Regarding specificity of US findings to fibrosis versus other liver pathology^13^, results indicated absence of steatosis. This indicates the sonographic changes observed here were not part of steatosis but were due to fibrosis. While echogenicity does increase with steatosis, the accompanying changes in heterogeneity and anisotropy may help identify fibrosis more specifically. Our data was collected under controlled imaging conditions including a fixed time-gain-compensation. Normalization via measures such as HRI may account for changing imaging parameters. The HRI may also help assess longitudinal changes despite ultrasound gain settings varying between studies.

In conclusion, we found that computer-extracted features of B-mode US images consistently increased over time in a quantifiable manner as hepatic damage and fibrosis progressed. In clinical practice, these unbiased quantitative US measures could inexpensively and quickly provide a quantitative measurement of the extent of hepatic fibrosis. In the future, these techniques may be applied to strengthen the clinical diagnosis and assessment of hepatic fibrosis and chronic liver disease. Going forward, these findings need to be validated in human subjects. Specifically, it should be confirmed whether the features measured at high frequency in this study can also be reliably measured and utilized at clinical frequencies.

## Methods

### Animals

All animal studies and protocols were approved by the University’s Institutional Animal Care and Use Committee. All methods were performed in accordance with the relevant guidelines and regulations. Twenty-two male Wistar rats (procured at 350–400 g body weight from Charles River Laboratories) were acclimated in the housing facilities for one week. They then began a diet of 0.01% diethylnitrosamine (DEN) (Sigma Aldrich, St. Louis, MO, USA) in their drinking water, ingested ad libitum for 12 weeks. The DEN model has been well-developed as a model of fibrosis and hepatocellular carcinoma^47,48^. The changes and spectrum of disease in fibrogenesis have also been utilized in imaging studies to track the progression of fibrosis^49^. As a control group, four rats were not fed DEN in their drinking water. The rats were euthanized using carbon dioxide asphyxiation or died naturally after tumors had developed, 108–135 days from the start of the DEN or control diet. A necropsy was performed and tissue from lobes in the right and left sides of the liver was harvested for histologic examination.

### Image acquisition and analysis

**Figure 5:**
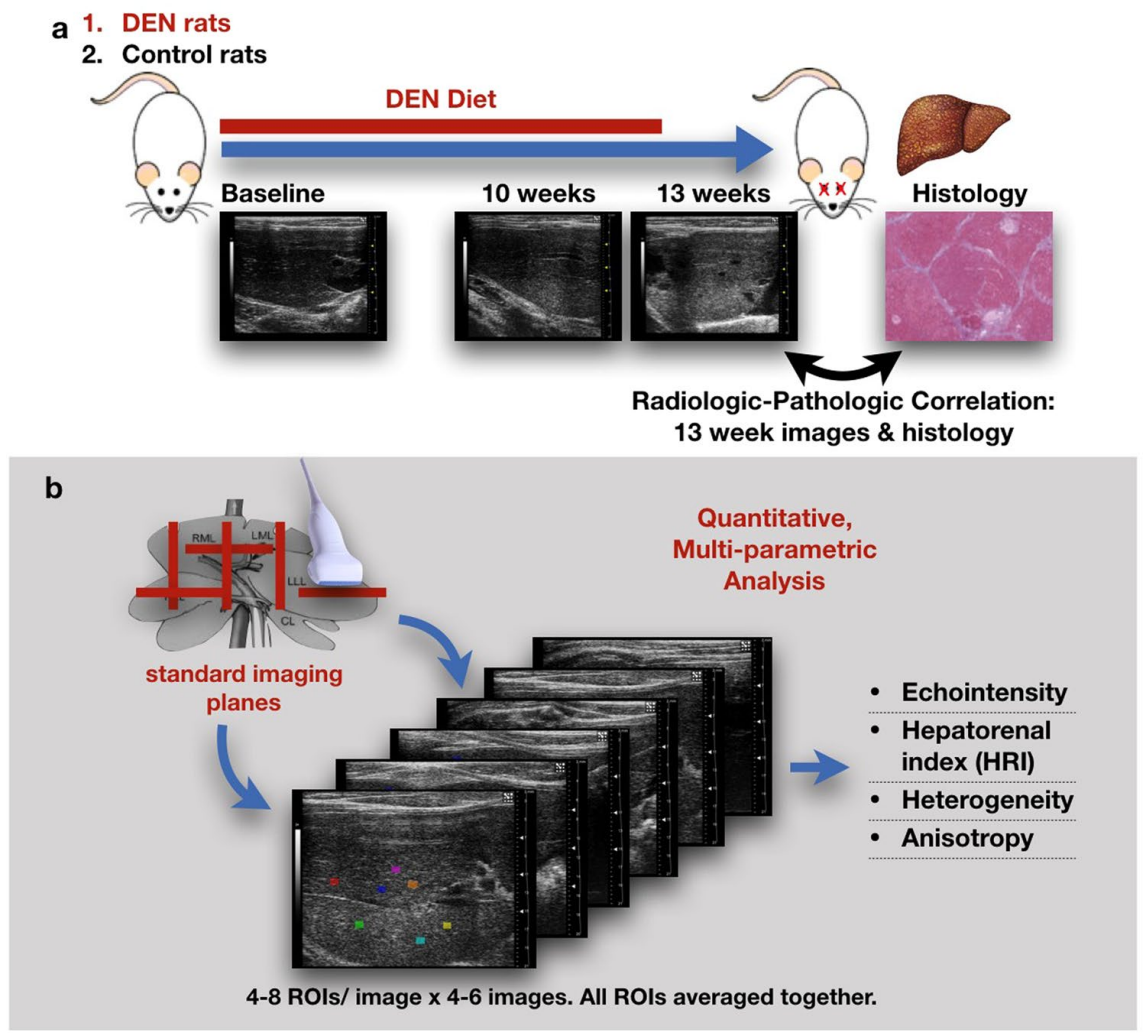
Study design and technique description. (**a**) Diagram of rodent studies. The treated rats were fed 0.01% diethylnitrosamine (DEN) for 12 weeks but it was not administered to the control group. B-mode hepatic ultrasound were made at 0 (baseline), 10 and 13 weeks, after which the rats were euthanized and liver samples were submitted for histologic examination; the histologic features were correlated with the 13 weeks ultrasound findings. (**b**) At each time point, 4–6 B-mode hepatic ultrasound images were acquired in standard imaging planes. 4–8 ROIs were placed on each image and analyzed for the listed textural features.

Sonographic images (Visualsonics VevoLAZR, Fujifilm, Toronto, Canada) of each rat were acquired at three time points: before diet initiation (0 weeks) and 10 and 13 weeks after the start of DEN administration (Fig. 5). Four to six B-mode images were acquired in standard transverse and sagittal imaging planes of the right and left lobes of the liver and the right liver-kidney interface. Imaging presets (gain = 18 dB, high sensitivity, 100% power, transmit frequency 21 MHz, and high line density) and time compensation gain were optimized and standardized. Four to eight regions of interest (ROI) were selected manually by a trained radiologist (LRS) to ensure comprehensive inclusion of multiple representative parts of the liver parenchyma and exclusion of imaging artifacts such as acoustic shadowing, enhancement, or reverberation. Intraobserver analysis was conducted on the same images with new ROI placement after more than one month, ensuring lack of memory bias. ROIs were analyzed offline using MaZda software (V 4.6, Technical University of Lodz: Lodz, Poland) for echointensity (brightness level), heterogeneity (local variance within ROIs), anisotropy (regional variance between ROIs), and hepatorenal index (HRI; brightness normalized to renal cortex)^50^. The ROI values for each parameter were averaged for each rat.

The features extracted from each ROI are statistical computations that represent the real texture of the tissue. The tissue’s nondeterministic properties govern the distributions and relationships between the gray levels of an image, which are then analyzed by the Mazda software. Echointensity and heterogeneity features are computed directly from the intensity of the pixels, taken from the mean intensity and standard deviation of intensity. Anisotropy is the calculated standard deviation of the echointensity between the ROIs in all the planes measured throughout the liver. Hepatorenal index is calculated from the mean of three ROIs placed in the hepatic tissue adjacent to the right kidney divided by the mean of three ROI placed in the renal cortex.

Vascular measurements were measured from B-mode images obtained at baseline, 10 weeks and 13 weeks. Diameters of the right hepatic vein, inferior vena cava and portal vein were measured using Vevolab software (Visualsonics, Fujifilm, Toronto, Canada).

### Histopathologic analysis

At necropsy, the liver lobes were assessed for fibrosis and nodularity on gross pathology. Portions of non-tumorous liver were preserved in 10% phosphate-buffered formalin and transferred to 50% ethanol after 48–72 hours and then embedded in paraffin and processed for histological examination with hematoxylin and eosin (H&E) and trichrome staining. Each histologic section was graded according to METAVIR scoring system. For animals in which multiple grades of fibrosis were identified, the highest-grade present was used to characterize the severity of fibrosis for that rat. Sonographic-pathologic correlations were made by correlating the imaging features at final the time point (13 weeks) with the METAVIR score.

### Statistics

Imaging features (echointensity, HRI, heterogeneity, anisotropy, and vascular measurements) in the DEN and control groups were assessed for a significant change over the three time points using a repeated measures ANOVA. The Greenhouse-Geisser correction for sphericity was used. Subsequent pairwise two-tailed Student’s t-tests using the least significant difference method were used between timepoints for ANOVA tests showing a significant difference. A two-tailed t-test was also performed to look for differences between the treated and control groups at each time point. Paired Student’s t-tests between the initial and repeated values of tissue features were used for intraobserver analysis. Spearman correlations were made between the non-parametric semi-quantitative, ordinal data of the METAVIR scoring system and the numeric values of the sonographic analyses. An ordinal multiparametric logistic regression was carried out with all four features and the METAVIR score. A stepwise backward selection with 0.2 significance for removal and 0.1 significance for addition was used to select which features best correlated with the histologic score. An alpha of 0.05 was considered statistically significant. Statistics were calculated using Stata version 15.0, StataCorp, College Station, TX^51^.

## Supplementary information

Supplementary file
