# Supplementary material for: B-mode ultrasound for the assessment of hepatic fibrosis: a quantitative multiparametric analysis for a radiomics approach
Source: Sci Rep. 2019 Jun 18;9:8708. doi: 10.1038/s41598-019-45043-z (PMC6581954; doi:10.1038/s41598-019-45043-z)
Supplement: Supplementary file 1 — Supplementary file [file 41598_2019_45043_MOESM1_ESM.docx]

**Supplemental Information**

**B-mode ultrasound for the assessment of hepatic fibrosis: a quantitative multiparametric analysis for a radiomics approach**

Julia C. D’Souza^1,2*^, Laith R. Sultan^1*^, Stephen J. Hunt^1,2,^ Susan M. Schultz^1^, Angela K. Brice^3,4^, Andrew K. Wood^4^, Chandra M. Sehgal^1^

**SI. Table 1: Sonographic feature comparison between first and second analyses**

|  |  | DEN | | | | Control | | |  |
| --- | --- | --- | --- | --- | --- | --- | --- | --- | --- |
|  |  | | **0 wk** | **10 wk** | **13 wk** | **0 wk** | **10 wk** | **13 wk** | |
| Echointensity | **A1** | | *37.1* | 53.5 | 57.7 | 35.1 | 35.4 | 33.2 | |
|  | **A2** | | *36.0* | 55.7 | 55.8 | 30.1 | 35.7 | 33.7 | |
|  | **p** | | *p=0.*35 | *p=* *0.31* | *p=* *0.31* | *p=0.*26 | *p= 0.94* | *p=0.72* | |
| Heterogeneity | **A1** | | 208.7 | 344.6 | 376.8 | 245.4 | 214.9 | 216.6 | |
|  | **A2** | | 215.7 | 390.8 | 358.8 | 164.2 | 221.3 | 187.8 | |
|  | **p** | | *p=* *0.57* | *p=* *0.051* | *p=* *0.40* | *p=* *0.053* | *p=* *0.83* | *p=* *0.08* | |
| HRI | **A1** | | 0.280 | 0.458 | 0.527 | 0.263 | 0.225 | 0.246 | |
|  | **A2** | | 0.287 | 0.476 | 0.542 | 0.246 | 0.312 | 0.241 | |
|  | **p** | | *p=0.65* | *p=0.50* | *p=0.63* | *p=0.69* | *p=0.31* | *p=0.81* | |
| Anisotropy | **A1** | | 11.57 | 15.3 | 16.56 | 8.95 | 8.98 | 8.94 | |
|  | **A2** | | 8.94 | 14.1 | 12.38 | 9.20 | 9.28 | 5.93 | |
|  | **p** | | *p=* *0.0007* | *p=* *0.34* | *p= 0.0001* | *p=* *0.85* | *p=* *0.87* | *p=* *0.14* | |

*A1= analysis 1, A2 = Analysis 2, p = p-values from paired Student’s T-test

**SI. Table 2: Agreement of F0/F1 samples with larger baseline sample of healthy tissue**

|  | F0/F1 13-week (n = 4) | Baseline (n = 22) | P-value (Baseline vs F0/F1) |
| --- | --- | --- | --- |
| Echointensity | 33.2 ± 2.0 | 37.1 ± SD 7.8 | 0.34 |
| Heterogeneity | 216.6 ± 25.5 | 208.7 ± 58.3 | 0.79 |
| HRI | 0.25 ± 0.05 | 0.28 ± 0.06 | 0.29 |
| Anisotropy | 8.9 ± 1.6 | 11.6 ± 3.0 | 0.10 |

**SI. Figure 1: Size characteristics of liver and fibrosis microstructure in humans versus rats**

|  | **Human** | **Rat** |
| --- | --- | --- |
| **Hepatocyte** | 20-30 um; 15 um each side nearly cuboidal (Lodish, H. 2000) | 13-38 um, 10-26 um width  (Engleman 1981)  17 um (Weibel 1969) |
| **Sinusoid** | Portal and septal sinusoids in average are longer by 205 and 124 um (Teutsch 2005) |  |
| **Acini** | Width of an acinus is twice the length of a sinusoid | 250 um (Rappaport, et al. 1957) |
| **Fibers  (fibrotic septae)** | 11.8 - 157.3 um (Wang 2018) | 7.6 - 80.2 um, median 18.8 um (study measurements) |
